# Global sensitivity of EEG source analysis to tissue conductivity uncertainties

**DOI:** 10.3389/fnhum.2024.1335212

**Published:** 2024-03-12

**Authors:** Johannes Vorwerk, Carsten H. Wolters, Daniel Baumgarten

**Affiliations:** ^1^Institute of Electrical and Biomedical Engineering, UMIT TIROL—Private University for Health Sciences and Health Technology, Hall in Tirol, Austria; ^2^Institute for Biomagnetism and Biosignalanalysis, University of Münster, Münster, Germany; ^3^Otto Creutzfeldt Center for Cognitive and Behavioral Neuroscience, University of Münster, Münster, Germany

**Keywords:** EEG, forward modeling, finite element method, source analysis, sensitivity analysis, uncertainty quantification

## Abstract

**Introduction:**

To reliably solve the EEG inverse problem, accurate EEG forward solutions based on a detailed, individual volume conductor model of the head are essential. A crucial—but often neglected—aspect in generating a volume conductor model is the choice of the tissue conductivities, as these may vary from subject to subject. In this study, we investigate the sensitivity of EEG forward and inverse solutions to tissue conductivity uncertainties for sources distributed over the whole cortex surface.

**Methods:**

We employ a detailed five-compartment head model distinguishing skin, skull, cerebrospinal fluid, gray matter, and white matter, where we consider uncertainties of skin, skull, gray matter, and white matter conductivities. We use the finite element method (FEM) to calculate EEG forward solutions and goal function scans (GFS) as inverse approach. To be able to generate the large number of EEG forward solutions, we employ generalized polynomial chaos (gPC) expansions.

**Results:**

For sources up to a depth of 4 cm, we find the strongest influence on the signal topography of EEG forward solutions for the skull conductivity and a notable effect for the skin conductivity. For even deeper sources, e.g., located deep in the longitudinal fissure, we find an increasing influence of the white matter conductivity. The conductivity variations translate to varying source localizations particularly for quasi-tangential sources on sulcal walls, whereas source localizations of quasi-radial sources on the top of gyri are less affected. We find a strong correlation between skull conductivity and the variation of source localizations and especially the depth of the reconstructed source for quasi-tangential sources. We furthermore find a clear but weaker correlation between depth of the reconstructed source and the skin conductivity.

**Discussion:**

Our results clearly show the influence of tissue conductivity uncertainties on EEG source analysis. We find a particularly strong influence of skull and skin conductivity uncertainties.

## 1 Introduction

Electroencephalography (EEG) is a frequently used tool for functional brain imaging in both research and clinical care (Brette and Destexhe, 2012). A huge advantage of EEG over, e.g., functional magnetic resonance imaging (fMRI), is its time resolution in the millisecond range. To localize the brain activity underlying a measured signal it is necessary to solve the EEG inverse problem (Knösche and Haueisen, 2022). As a prerequisite for solving the EEG inverse problem, it is necessary to model the propagation of the electric fields evoked by brain activity through the head tissues, which are measured as the EEG signal at the head surface (EEG forward problem). Accurately solving the EEG forward problem is one important factor to reliably solve the EEG inverse problem (others are, e.g., the choice of an adequate inverse method).

The EEG forward problem is commonly solved using numerical methods, such as the boundary element method (BEM; Kybic et al. 2005) or the finite element method (FEM; Yan et al. 1991; Buchner et al. 1997), and, therefore, requires a discretized volume conductor model of the head, i.e., a 3d representation of the head distinguishing the different conductive tissues. It was shown that the use of accurate, individual head models distinguishing five or more tissues (skin, skull, cerebrospinal fluid/CSF, gray matter, white matter) is important to obtain accurate EEG forward solutions (Vorwerk et al., 2014; Nielsen et al., 2023), which, in consequence, are essential for accurate EEG inverse solutions (Ramon et al., 2006; Cho et al., 2015; Neugebauer et al., 2017; Asadzadeh et al., 2020; Azizollahi et al., 2020). However, besides the geometrical accuracy of the head model, also the values chosen for the tissue's electrical conductivities influence the obtained EEG forward solution. Neglecting interindividual variations of these conductivities in the computation of the EEG forward solution may therefore lead to inaccurate EEG inverse solutions (Vanrumste et al., 2000; Chen et al., 2010; Akalin Acar and Makeig, 2013; Aydin et al., 2014; Vorwerk et al., 2019a). Such interindividual variations may, e.g., occur due to age or disease state (Akhtari et al., 2002; McCann et al., 2019; Antonakakis et al., 2020). Conductivity calibration based on electrical impedance tomography (EIT), EEG, or combined EEG/MEG has been proposed as a means to alleviate the influence of conductivity uncertainties (Huang et al., 2007; Acar et al., 2016; Fernández-Corazza et al., 2017). Most of these studies focused on fitting the skull conductivity, but it is unclear whether the dependency on the skull conductivity is similarly strong for all source positions and whether fitting the skull conductivity is thus always the optimal choice.

Sensitivity studies allow estimating to what extent variations of the tissue conductivities influence the results of EEG forward solutions. So far, studies found that variations of skin and skull conductivities have the strongest influence for the EEG (Gençer and Acar, 2004; Vallaghé and Clerc, 2008; Vorwerk et al., 2019a). However, to the best of our knowledge, existing EEG sensitivity studies only investigated a few source positions that were assumed to be representative. Especially in highly-detailed head volume conductor models, as they are more and more frequently used nowadays (Buzzell et al., 2017; Piai et al., 2017; Staljanssens et al., 2017; Gao et al., 2019; Zaky et al., 2023), the choice of the source positions might have a strong influence on the results of the sensitivity analysis.

In this study, we investigate the sensitivity of EEG forward solutions to conductivity variations for sources distributed over the whole cortex surface. Furthermore, we investigate the sensitivity of EEG inverse solutions to the same conductivity variations, and determine to what extent changes of the EEG inverse solution correlate with the sensitivity of the EEG forward solutions to tissue conductivity variations.

## 2 Materials and methods

### 2.1 Head model

**Figure 1:**
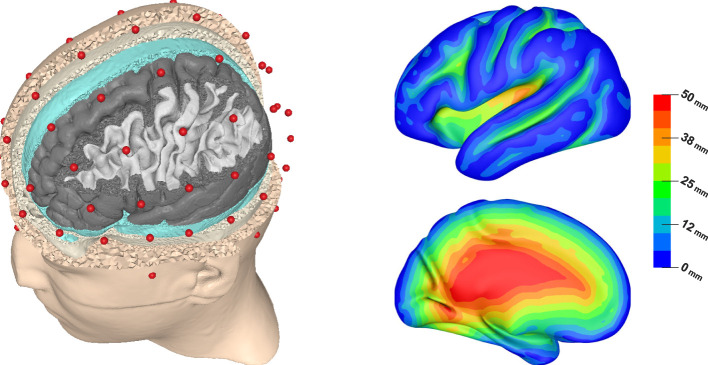
Visualization of the FEM head model showing electrode positions (red) and (from outside to inside) skin, skull, CSF, gray matter, and white matter surfaces **(left)**. Lateral and medial view of source depth (distance to inner skull surface) visualized on inflated left cortex surface **(right)**.

We generated a head model based on the segmentations provided for the *New York Head* (https://www.parralab.org/nyhead/). The segmentations of brain and non-brain tissues are based on the symmetric ICBM-152 v2009 and the symmetric ICBM-152 v6 average atlases, respectively (https://nist.mni.mcgill.ca/atlases/), whereas the lower parts of the head are from a separate segmentation (Huang et al., 2016). We slightly modified the segmentations to ensure a minimal thickness of the gray matter of 2.5 mm. Furthermore, we reduced the number of tissue compartments to five (white matter, gray matter, CSF, skull, skin). We used SimNIBS 4 (https://simnibs.github.io/simnibs/; Puonti et al. 2020) for head mesh generation and to obtain gray matter, white matter, and central cortex surfaces for both hemispheres that will be used for source space construction and visualization. We chose to generate an especially fine mesh structure in the gray and white matter volumes; the resulting tetrahedral head mesh consisted of 3,473,632 nodes and 20,703,247 elements (see Figure 1, left). We used the electrode positions provided with the New York Head to create a realistic sensor configuration corresponding to a 10-10 layout, resulting in 80 electrode positions.

### 2.2 Source spaces and EEG forward simulations

We created the source space for this study based on the central surface of the cortex obtained from SimNIBS, which is the estimated surface in the middle of gray matter/CSF and gray/white matter boundaries. It has to be observed that this central surface represents a closed surface for each hemisphere, i.e., the hemispheres are split at the corpus callosum. Furthermore, these surfaces also cover some deep brain regions that could be attributed to subcortical brain structures such as the thalamus or the basal ganglia, whereas brainstem and cerebellum are excluded. Due to the symmetry of the underlying segmentation, we only considered the left hemisphere. For reasons of computational efficiency, we downsampled the surface to 34,997 vertices.

To achieve high numerical accuracy in our forward simulations, we ensured that for all source positions the closest node of the head mesh is fully contained in the gray matter compartment, i.e., all mesh elements this node is part of have to belong to the gray matter compartment (Vorwerk et al., 2019b). Source positions for which this was initially not the case were shifted toward the closest node fully contained in the gray matter compartment until this condition was fulfilled. For each source position, we calculated the surface normal as a physiologically plausible source direction at this position. We refer to this source space as *sources_cortex*. For visualization purposes, we created an inflated version of the central cortex surface underlying this source space.

To avoid an inverse crime when evaluating the sensitivity of EEG source analysis to conductivity uncertainties, we created a second source space based on the dual mesh of the source space *sources_cortex*. This means that the source positions for this second source space are the triangle centers of the cortex surface on which the original source space *sources_cortex* is based. The resulting source space consists of 69,990 vertices; we refer to this source space as *sources*_cortex^*^. *sources*_cortex^*^ is used for all inverse calculations, whereas *sources_cortex* is used for the forward simulations. Again, we ensured that the closest node of the volume conductor model for all source positions of *sources*_cortex^*^ is fully contained in the gray matter compartment. On average, the distance between a node of *sources*_cortex^*^ and the closest node in *sources_cortex* is 0.8 mm, which is the average minimal localization error, accordingly.

We used the FEM multipole approach for all forward simulations, as it was shown to achieve high numerical accuracy with a high computational efficiency (Vorwerk et al., 2019b). The multipole approach was implemented based on the FieldTrip-SimBio pipeline (Vorwerk et al., 2018).

### 2.3 EEG forward problem sensitivity analysis

We mostly rely on Monte Carlo approaches for our sensitivity analysis. To handle the large number of forward simulations for different conductivity values necessary for the sensitivity/uncertainty analysis, we employ generalized polynomial chaos (gPC) expansions (Vorwerk et al., 2019a). Based on predefined probability distributions and precomputed forward solutions generated for corresponding sets of conductivities, gPC expansions allow to rapidly approximate accurate forward simulations for arbitrary conductivity values. We used UQLab 2.0 to perform the gPC calculations in this study (https://www.uqlab.com/; Marelli and Sudret 2014). The details of the used gPC approach are described in Vorwerk et al. (2019a).

As in Vorwerk et al. (2019a), we chose uniform distributions for all tissue conductivities considered uncertain. The uniform distribution represents minimal knowledge about the distribution of these conductivities. The intervals within which each conductivity could vary are shown in Table 1; the CSF conductivity was not considered uncertain as it was shown to have a negligible inter-individual variation (Baumann et al., 1997).

**Table 1:** Tissue conductivity intervals (mS/m).

**Tissue**	**Min. σ_*min*_**	**Max. σ_*max*_**	**Standard σ_*st*_**	**References**
Skin	280.0	870.0	430.0	Haueisen et al., 1997; Ramon et al., 2004
Skull	1.6	33.0	10.0	Akhtari et al., 2002; Hoekema et al., 2003; Dannhauer et al., 2011
CSF	1,769.6	1,810.4	1,790.0	Baumann et al., 1997
GM	220.0	670.0	330.0	Haueisen et al., 1997; Ramon et al., 2004
WM	90.0	290.0	140.0	Haueisen et al., 1997; Ramon et al., 2004

With four tissue conductivities varying uniformly within the ranges indicated in Table 1, it is of interest to estimate the contribution of each of the four uncertain tissue conductivities to the overall variation of the EEG forward solution. Therefore, we use Sobol indices (Sobol, 2001). These are defined as


(1) $$\begin{array}{l} S_{i_{1},\ldots ,i_{s}}=\frac{𝕍\left(i_{1},\ldots ,i_{s}\right)}{𝕍}, \end{array}$$

i.e., the Sobol index *S*_*i*_1_, …, *i*_*s*__ is defined as the ratio between the variance caused by the interaction of the subset of input parameters {*i*_1_, …, *i*_*s*_}⊂{1, …, *n*} and the overall variance. It is important to note that 𝕍(*i*_1_, …, *i*_*s*_) only includes the variance caused by the interaction of the subset of indices *i*_1_, …, *i*_*s*_ but not the contributions that can be attributed to a single variable or a smaller subset of these variables. For example, for a second-order Sobol index *S*_*i, j*_, the variance 𝕍(*i, j*) does not include the variances 𝕍(*i*) and 𝕍(*j*) that can be attributed to either *i* or *j* individually.

In this study, we focus on first- and second-order Sobol indices, i.e., the share of variance caused by the uncertainty of a single uncertain tissue conductivity or the share of variance caused by the uncertainty of two conductivities that cannot be attributed to the uncertainties of a single conductivity, respectively. We further consider total-effect or full Sobol indices, $S_{i}^{T}$, for which all Sobol indices involving a certain input parameter *i* are summed up:


(2) $$\begin{array}{l} S_{i}^{T}=S_{i}+\underset{i\neq j}{\sum }S_{i,j}+\underset{\begin{array}{c} i\neq j,k\\ j<k \end{array}}{\sum }S_{i,j,k}+\ldots . \end{array}$$

The Sobol indices were computed with UQLab using a Monte Carlo approach with 50,000 samples per parameter. We found that this number of samples guaranteed a more than sufficient convergence of the Sobol indices for the requirements of this study. To calculate the Sobol indices, the *Janon estimator* was used (Janon et al., 2014, Equation 2.6), which has optimal asymptotic variance and is robust against model perturbations. Further details regarding the calculation of the Sobol indices are provided as Supplementary material. Furthermore, we would like to refer the interested reader to the original publication of Janon et al. (2014) or the UQLab User Manual (https://uqftp.ethz.ch/uqlab_doc_pdf/2.0.0/UserManual_Sensitivity.pdf) for additional information.

In the sensitivity analysis of EEG forward simulations, we have the challenge that we do not have a single output parameter, but each computed electrode potential is a separate output parameter. To allow for a comprehensible and easily interpretable evaluation of the Sobol indices, we introduce the relative difference measure (RDM) and the magnitude error (MAG) (Meijs et al., 1989). Computing these error measures in comparison to a reference solution, it is possible to express the topography and magnitude change of the set of electrode potentials through a single parameter for each source position. Interpreting RDM and MAG as functions of the conductivities, we can then compute the Sobol indices for the changes of RDM and MAG, expressing the influence of changes in each tissue conductivity on signal topography and magnitude. A similar approach was previously used by Vallaghé and Clerc (2008). As a reference solution, we use the forward solution for the standard conductivity values indicated in Table 1.

RDM and MAG are defined as follows:


(3) $$\begin{array}{l} RDM(u^{test},u^{ref})\;=\;{\left\|\frac{u^{test}}{||u^{test}|{|}_{2}}-\frac{u^{ref}}{||u^{ref}|{|}_{2}}\right\|}_{2},\\ MAG(u^{test},u^{ref})\;=\frac{||u^{test}|{|}_{2}}{||u^{ref}|{|}_{2}}, \end{array}$$

where *u*^*test*^ corresponds to the vector of electrode potentials for varied conductivities and *u*^*ref*^ corresponds to the vector of electrode potentials for standard conductivities.

The RDM represents the change in signal topography in comparison to the reference solution, which was shown to be linked to source localization accuracy, whereas the MAG defines the change in signal magnitude. In most applications of EEG source analysis, only the change of signal topography is of relevance, whereas there are only a few cases where the exact source magnitude is of interest. Thus, we mainly focus on the RDM evaluations in this study.

### 2.4 EEG source analysis sensitivity analysis

To evaluate the influence of conductivity uncertainties on EEG inverse solutions, we performed forward simulations for 1,000 randomly drawn sets of conductivities. Following, for each source position, we calculate inverse solutions using the source space sources_cortex^*^ and a leadfield matrix obtained with standard conductivity values. This scenario corresponds to the common problem of EEG source analysis that the actual tissue conductivities that influence the measurement result are unknown, while the EEG source analysis is performed using conductivity values from the literature. The resulting 1,000 source localizations per source position can then be evaluated to investigate the sensitivity of the EEG inverse solution to conductivity variations.

As an inverse method, we used goal function scans (GFS) with a free source orientation, i.e., the source position *i* in the source space for which


(4) $$\begin{array}{l} GoF=1-{\left(\frac{||u_{\text{meas}}-L_{i}L_{i}^{+}u_{\text{meas}}|{|}_{2}}{||u_{\text{meas}}|{|}_{2}}\right)}^{2} \end{array}$$

is maximal is selected as the reconstructed source location. Here, *u*_meas_ is the (simulated) measurement result, ||·||_2_ is the Euclidian norm, *L*_*i*_ = *L*(**x**_*i*_) is the *#sensors*×3 leadfield matrix for position **x**_*i*_, i.e., a matrix containing the forward simulation results for dipoles with moments oriented in each of the three cartesian directions at the source position, and $L_{i}^{+}$ its Moore-Penrose inverse. In a single dipole scenario, as it is given in our simulation study, the GFS reliably finds the source position that optimally explains the data (Knösche, 1997; Fuchs et al., 1998).

To evaluate the influence of the conductivity uncertainties on the source localization, we calculate and visualize the average localization error, i.e., the distance between source localization and original source position, for the 1,000 sets of conductivities at each source position. This allows to understand how much the conductivity variations affect the accuracy of the source localization for each source position. We further calculate and visualize the ratio between the difference in source depth and the localization error and again take the average over all sets of conductivities to analyze to what extent the localization error can be explained by a change in source depth. Here, “change in source depth” denotes the absolute value of the difference between the source depth of the original source and the source depth of the reconstructed source.

To understand the influence of the variation of each tissue conductivity on the source localization, we calculate and visualize the correlation between deviations of each conductivity from the average conductivity and distance of the source reconstruction to the center of the point cloud of source localizations, and the correlation between each conductivity and the source depth. Again, these measures are calculated for each source position in *sources_cortex*.

The Sobol indices computed as described in Section 2.3 only indicate which tissue conductivities contribute most to the variation of an output parameter, but not how strongly this output parameter varies overall. To understand the dependency between RDM and source analysis accuracy, we calculate the RDM for all source positions and all 1,000 considered sets of conductivities, and visualize the average RDM and the correlation between localization error and RDM for each position in source space *sources_cortex*.

### 2.5 Evaluation

We employ two kinds of evaluation in this study. On the one hand, we visualize the results directly on an inflated cortex surface. This allows to visually identify the most affected brain areas. On the other hand, we plot the median Sobol indices and the corresponding 50% confidence interval, i.e., the interval between upper and lower quartile, as a function of the source depth. In this case, the source depth is calculated as the distance from the source position to the inner skull surface. These plots allow to identify in how far the source depth affects the sensitivity of the forward solution toward the different conductivities. Similar plots are also created for the correlation between the tissue conductivities and source localization error/source depth as a function of the source depth.

Figure 1 (right) allows to understand the distribution of source depths, which is necessary to interpret these plots. Unlike in spherical models, there is no unique definition of source depth in realistically shaped head models. In this study, we chose to define source depth as the distance of a source position to the inner skull surface. We chose this definition over the also frequently used distance to the outer skin surface, as it led to better interpretable results when plotting effect measures as a function of source depth. In result, some source positions that would usually be considered as “deep”, e.g., in the medial temporal lobe, are classified as rather superficial in our study, as they are close to the base of the skull. Furthermore, Figure 1 (right) shows that source depths smaller than 5 mm correspond to sources on top of gyri, which can be assumed to mostly have a quasi-radial orientation. An exception are sources at the inferior surfaces of frontal and temporal lobe, which have a rather quasi-tangential orientation. Sources up to a depth of 30 mm correspond to sources inside of sulci, which are assumed to be mostly located on sulcal walls and have a quasi-tangential orientation in consequence. However, for source depths of about 15–30 mm this also includes sources at the bottom of sulci, which again have a rather quasi-radial orientation. Sources at depths of 30 mm and deeper mostly correspond to source positions in the insula, the longitudinal fissure and subcortical regions.

As our plots are based on the median and upper and lower quartile, the results should be stable against outliers and especially the median should represent the results for the dominant type of sources at each source depth well. This would correspond to quasi-radial sources for source depths smaller than 5 mm, quasi-tangential sources for source depths of about 5–30 mm, and sources in the insula and the longitudinal fissure for larger source depths.

## 3 Results

### 3.1 EEG forward problem

#### 3.1.1 Signal topography

**Figure 2:**
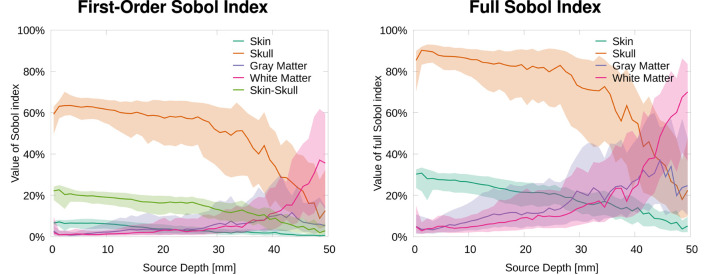
Median and 50% confidence interval of first-order and skin-skull second-order **(left)** and full **(right)** Sobol indices for signal topography/RDM plotted as a function of source depth.

In this section, we analyze the sensitivity of the topography of EEG forward solutions toward tissue conductivity variations. Therefore, we calculated the Sobol indices of the RDM in comparison to a reference solution (see Equation 3). Figure 2 shows that the skull conductivity clearly has the strongest influence on the signal topography for nearly all source depths. Looking at the first-order and the second-order skin-skull interaction Sobol indices (Figure 2, left), we find a median skull conductivity Sobol index of about 60% for source positions with a depth of up to 35 mm, which includes basically all source positions except those deep in the longitudinal fissure and in subcortical regions (see Figure 1, right). Besides, we also find a strong influence of the skin-skull interaction for rather superficial sources. The median of this second-order Sobol index is at about 20% for the most superficial sources and gradually decreases for deeper sources. All other Sobol indices are below 10% for superficial and medium-deep sources. For sources deeper than 35 mm, e.g., sources deep in the longitudinal fissure, the sensitivity toward the white matter conductivity clearly increases, whereas the skull and skin-skull Sobol indices decrease.

The full Sobol indices (Figure 2, right), i.e., the sum of all variations attributed to one parameter (see Equation 2), underline the dominant influence of the skull conductivity for all sources that are not very deep even more. The median full skull conductivity Sobol index is higher than 80% for source depths smaller than 30 mm. As a result of the skin-skull conductivity interaction, also the full skin conductivity Sobol index is significant at a value of 30% for the most superficial sources. For deep sources, again, the sensitivity toward the white matter conductivity clearly increases.

**Figure 3:**
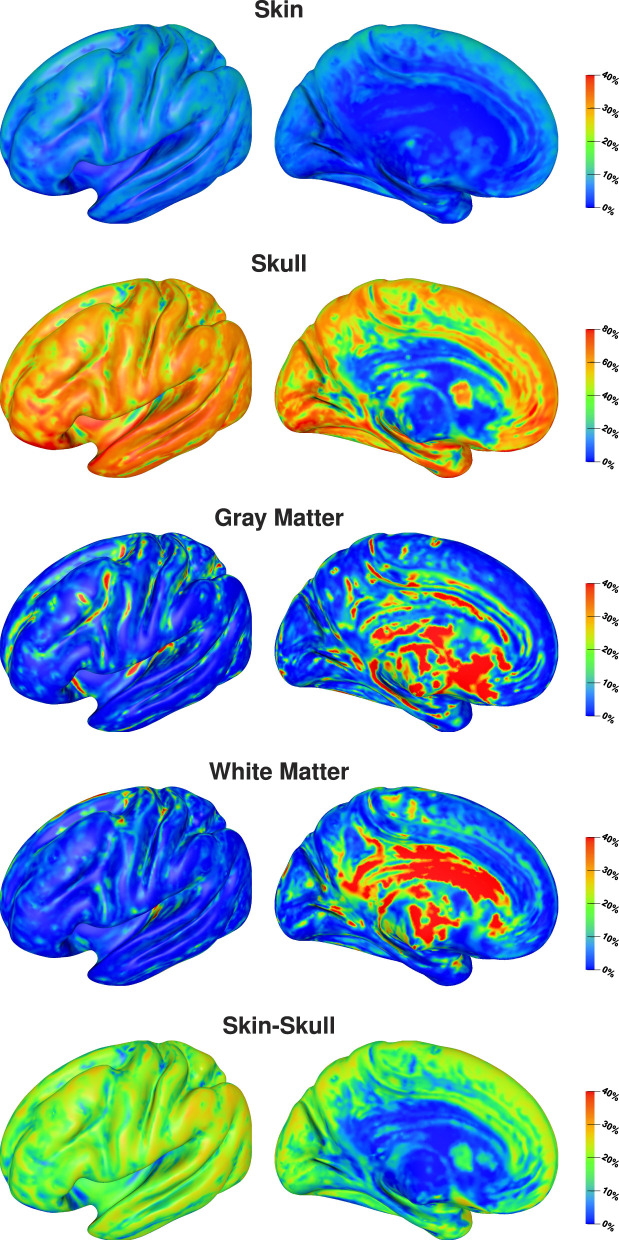
First-order and skin-skull second-order Sobol indices for signal topography visualized on inflated cortex surface; (fronto-)lateral **(left column)** and medial **(right column)** view. Please observe the different scalings of the colorbar.

The visualization of first- and second-order Sobol indices on the cortex surface underlines the influence of the skull conductivity (Figure 3). For large parts of the cortex surface, the Sobol index for the skull conductivity is clearly above 60% (Figure 3, second row). Lower Sobol indices are mainly found on top of gyri and at the bottom of sulci where the Sobol index drops to about 40%. Due to the choice of source orientations normal to the cortex surface, these source positions correspond to quasi-radial sources. Furthermore, the medial view shows small Sobol indices for source positions deep in the longitudinal fissure. For the skin-skull second-order Sobol index, we find sensitivities of about 30% for very superficial sources, whereas the sensitivity gradually decreases for deeper sources. For the skin conductivity, we find generally rather low Sobol indices of around 10% and lower. Here, lower values are especially found for deeper sources at the bottom of sulci and in some areas on top of gyri; higher values are consistently found on sulcal walls. For gray and white matter conductivities, the Sobol indices are almost zero for large parts of the cortex surface. However, strong outliers are found especially on gyral crowns and at sulci bottoms, where these Sobol indices are clearly increased. This corresponds to the positions for which the skull conductivity Sobol index was decreased and where we assume quasi-radial sources. Furthermore, we find increased Sobol indices for gray and white matter conductivities deep in the longitudinal fissure and in subcortical regions as can be seen in the medial view.

**Figure 4:**
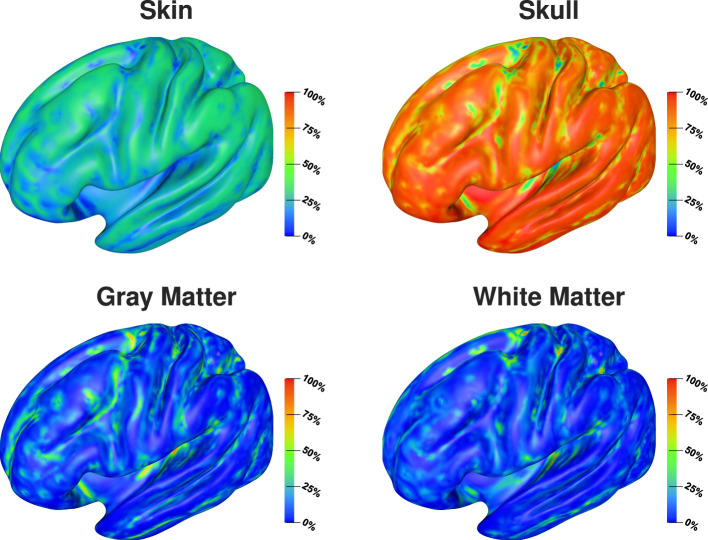
Full Sobol indices for signal topography visualized on inflated cortex surface; (fronto-)lateral view.

Visualizing the full Sobol indices (Figure 4), the predominance of the sensitivity toward the skull conductivity gets even more clear. Only for a few areas this sensitivity drops below 75%. Due to the addition of the skin-skull Sobol index, also the full skin Sobol index has a value of around 50% for large parts of the cortex surface. We especially find a notable decrease in the sensitivity to the skin conductivity on top of gyri and at the bottom of sulci. Gray and white matter conductivities again only show a significant sensitivity in a few areas, such as some gyral crowns and sulci bottoms.

#### 3.1.2 Signal magnitude

**Figure 5:**
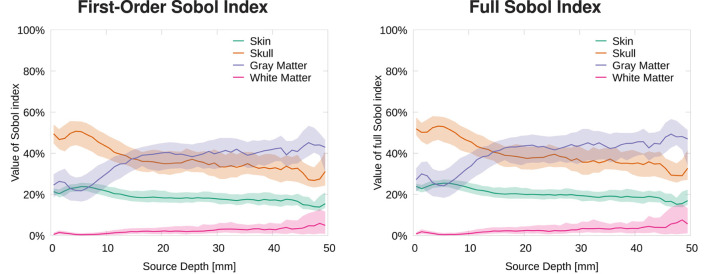
Median and 50% confidence interval of first-order **(left)** and full **(right)** Sobol indices for signal magnitude/MAG plotted as a function of source depth.

To evaluate the influence of conductivity uncertainties on the signal magnitude, we calculated the Sobol indices for the MAG (see Equation 3). Figure 5 shows the strongest influence for skull and gray matter conductivities. Furthermore, we observe that first-order and full Sobol indices are almost identical, as the higher-order interactions are negligible for the signal magnitude. Therefore, we only discuss the full Sobol indices here and also only provide the cortex plots for the full Sobol indices.

For the most superficial sources, we find the strongest influence for the skull conductivity with a Sobol index of about 50%. For the influence of the gray matter conductivity, we find a Sobol index of about 25% and for the skin conductivity of about 20%. The influence of the white matter conductivity is negligible and stays below 10% at all source depths. For slightly deeper sources of about 5 mm depth, the influence of skin and skull conductivity slightly increases, whereas that of gray matter slightly drops. With increasing source depth, the influence of the gray matter conductivity gradually increases up to a Sobol index of about 40% for sources with a depth of 20 mm and more, whereas the influence of skull and skin conductivities drops to Sobol indices below 40 and 20%, respectively, for sources with a depth of 20 mm and more.

**Figure 6:**
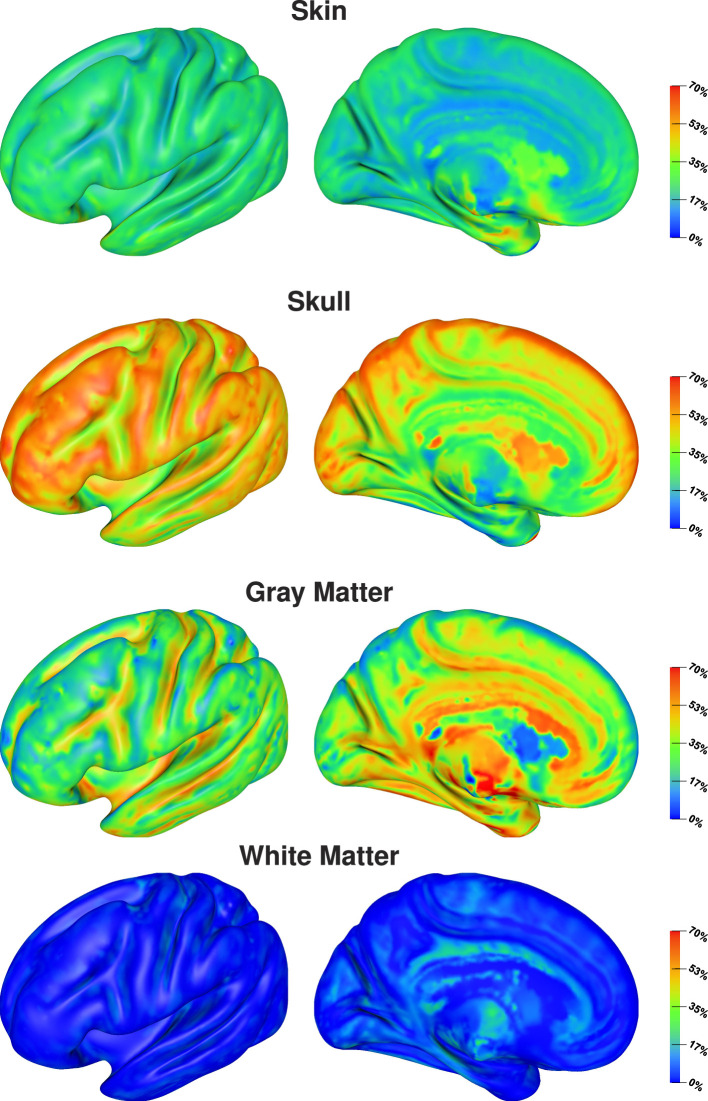
Full Sobol indices for signal magnitude visualized on inflated cortex surface; (fronto-)lateral **(left column)** and medial **(right column)** view.

The visualization on the cortex surface (Figure 6) shows the strongest influence of the skull conductivity on the signal magnitude for sources on top of gyri with Sobol indices above 50%. This influence gradually decreases to values around 35% for sources deeper inside the sulci. For the gray matter conductivity, we see the exact opposite with the weakest influence and Sobol indices of about 20–25% on top of gyri and a gradual increase toward values of up to 50% at the bottom of sulci and deep in the longitudinal fissure. For the skin conductivity, we find the same decrease from the top of gyri to the bottom of sulci as for the skull conductivity, just at a clearly reduced level with Sobol indices of about 25% and lower. For the white matter conductivity, we do not find a significant influence except for some deep brain regions visible in the medial view.

### 3.2 EEG inverse problem

Analyzing the influence of conductivity variations on EEG source analysis, we first focus on the general localization errors caused by simultaneous variations of all four tissue conductivities considered uncertain and analyze the direction of these localization errors, i.e., in how far these can be explained by an incorrect depth of the reconstructed source position. To understand the relationship between the results obtained in the forward simulation study, we further compare the distribution of the average localization error and the average RDM as well as the correlation between RDM and localization error at each source position. Subsequently, we analyze the correlation between localization errors and conductivity variations to understand which conductivities have the strongest influence on the localization errors.

**Figure 7:**
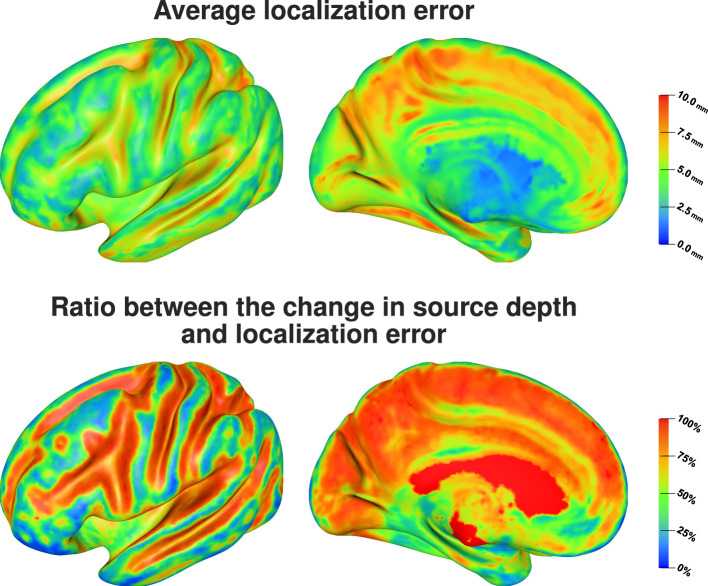
Average localization error **(top)** and the average ratio between the change in source depth and localization error **(bottom)** visualized on inflated cortex surface; (fronto-)lateral **(left column)** and medial **(right column)** view.

Figure 7 (top) shows that source positions inside the sulci are clearly more sensitive to localization errors due to conductivity uncertainties than superficial source positions on top of the gyri. We find average localization errors of up to 10 mm for sources deep inside of sulci, whereas the average localization errors remain below 5 mm for superficial sources. For rather superficial sources in the longitudinal fissure we find large localization errors as well, whereas the localization errors for deep brain regions that could be attributed to subcortical structures are small. Visualizing the ratio between change in source depth, i.e., the absolute value of the difference between depth of the original source position and depth of the reconstructed source position, and localization error, i.e., the distance between the original source position and the reconstructed source position, Figure 7 (bottom) shows that for the quasi-tangential sources on the sulcal walls and for sources in the longitudinal fissure the localization error is nearly completely caused by changes in source depth, whereas this is slightly less distinct for the quasi-radial sources at the bottoms of the sulci. For the quasi-radial sources on top of gyri, only a small fraction of the localization error can be explained by changes in source depth.

**Figure 8:**
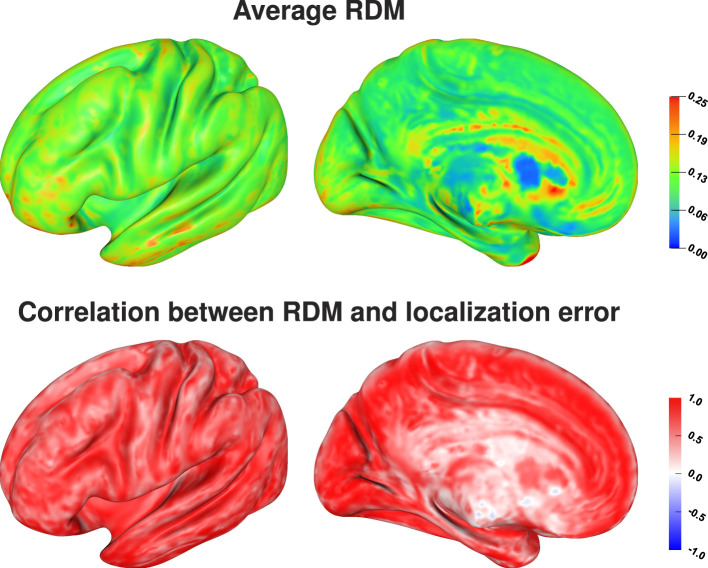
Average RDM **(top)** and correlation between RDM and localization error, i.e., distance between reconstructed and original source position, **(bottom)** visualized on inflated cortex surface; (fronto-)lateral **(left column)** and medial **(right column)** view.

Comparing the average localization error (Figure 7, top) and the average RDM for each source position (Figure 8, top) there seems to be no direct relation between the size of the topography errors at a source position due to conductivity variations and the resulting average localization error. The largest average RDMs are found on top of gyri, which are the source positions at which the average localization error is minimal. However, analyzing the correlation between RDM and localization error at each source position (Figure 8, bottom), we find a clear, positive correlation for the source positions for which we also find large localization errors (compare Figure 8, bottom, and Figure 7, top). For sources that are less affected by localization errors in general, we only find a weaker correlation between RDM and localization errors.

**Figure 9:**
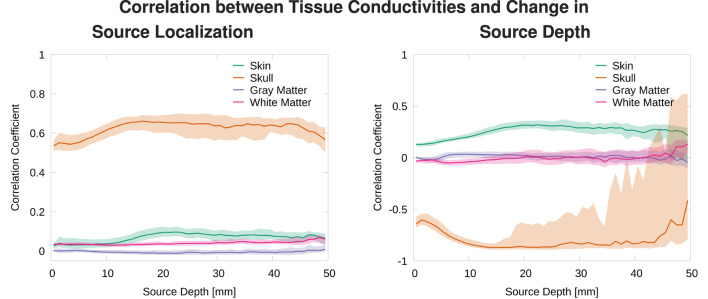
Median and 50% confidence interval of the correlation coefficient between deviation of tissue conductivities from mean, |σ_*i*_−(σ_*max*_−σ_*min*_)/2|, and distance to average source localization **(left)** and correlation coefficient between tissue conductivity and depth of localized source **(right)** plotted as a function of source depth.

To understand which tissue conductivities drive the overall localization errors and the changes in source depth, we calculated two different correlation coefficients. For the localization error, we calculated correlation coefficients between the absolute value of the deviation of a tissue conductivity from the mean conductivity for this tissue, |σ_*i*_−(σ_*max*_−σ_*min*_)/2|, and the distance of the source localization from the center of the point cloud of source localizations for each source position, ${\|x}_{i}-\frac{1}{n}\sum_{j}x_{j}|{|}_{2}$ (Figure 9, left). Taking the absolute value of the deviation was necessary here to be able to properly calculate a correlation, as the localization error can only be measured as a distance to a reference position (in our case the center of the point cloud of source localizations), i.e., the distance is always positive, regardless of whether the conductivity was increased or decreased. For the source depth, it was directly possible to calculate the correlation coefficients between tissue conductivities and depth of the corresponding reconstructed sources (Figure 9, right).

We find that changes in the skull conductivity have the by far strongest influence on localization errors, with a correlation coefficient of around 0.6 for all source depths. All other correlation coefficients have small values below 0.1 with the skin conductivity having the second highest correlation especially for sources deeper than 1 cm (Figure 9, top). We find a strong negative correlation between skull conductivity and source depth, especially for sources deeper than 1 cm. At the same time, we find a positive correlation of up to 0.3 between changes in skin conductivity and source depth. This means that using a higher skull conductivity for the simulated source leads to a more superficial source localization based on standard conductivities, whereas a higher skin conductivity leads to a deeper source localization. As shown in previous studies, changes in skin and skull conductivities have opposite effects and the effect of the skull conductivity is stronger.

We find an increasing variation in the correlation of skull conductivity and source depth for sources 20 mm and deeper. This is presumably caused by sources already being located relatively deep in sulci, for which a further increase in source depth within the sulci upon a decrease of the skull conductivity is not possible. These sources might then be mislocalized in a different brain structure but at a similar source depth, e.g., in a neighboring sulci, resulting in a reduced correlation coefficient.

**Figure 10:**
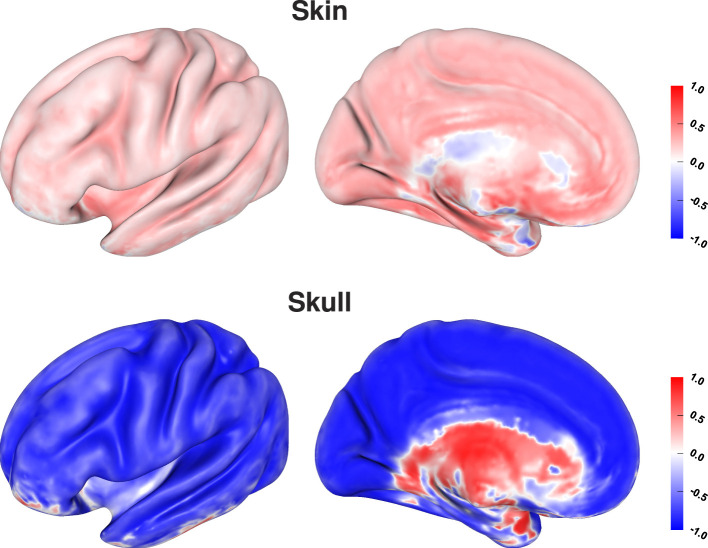
Correlation coefficient between tissue conductivity and depth of source localization for skin **(top)** and skull conductivity **(bottom)** visualized on inflated cortex surface; (fronto-)lateral **(left column)** and medial **(right column)** view.

Visualizing the correlation coefficients of skin and skull conductivities and source depth shows the strongest correlations for sources inside the sulci and especially on sulcal walls (Figure 10). This correlates to the source positions for which the localization error was mainly driven by an incorrect depth of the reconstructed sources (see Figure 7, bottom). The still relatively high correlation coefficients for sources on top of gyri can be of less relevance, since these source positions were previously found to be more robust against localization errors (see Figure 7, top), so the correlations were probably caused by rather small variations of the source localizations. For deep brain areas the medial view shows inverted correlation coefficients for both skin and skull conductivity. However, due to the generally small average localization errors in these areas (Figure 7, top) and the small influence of skin and skull conductivity on the signal topography for these sources (Figure 3) they presumably have only little influence in practice.

## 4 Discussion

### 4.1 EEG forward problem

In this study, we investigated the sensitivity of EEG forward and inverse solutions to conductivity uncertainties. Making use of Sobol indices, we found that variations of the skull conductivity have the by far strongest influence on the topography of EEG forward solutions (Figures 2–4). Furthermore, we found a notable influence of skin conductivity variations, especially through the second-order skin-skull interaction. For very deep sources (source depth of more than 40 mm), which corresponds to sources deep in the longitudinal fissure and in subcortical structures, the sensitivity to white and gray matter conductivity variations increases clearly. For the signal magnitude, we find a strong sensitivity to variations of skull and gray matter conductivities, with an especially strong influence of the skull conductivity for superficial, quasi-radial sources (Figures 5, 6). Furthermore, we find a notable influence of the skin conductivity which is almost constant for all source depths.

These results confirm the results of prior sensitivity studies (Gençer and Acar, 2004; Vallaghé and Clerc, 2008; Vorwerk et al., 2019a). Using realistic three- and four-layer models, Vallaghé and Clerc (2008) found the strongest sensitivity for the skin-skull interaction, whereas we found the strongest sensitivity for variations of the skull conductivity in our study with a clearly lower sensitivity to the skin-skull interaction. However, for EEG source analysis, the variations of skin and skull conductivity were shown to have almost identical effects on localization errors but with opposing directions, and the influence of the skull conductivity was found to be stronger than that of the skin conductivity (Figure 10, Vorwerk et al. 2019a). Thus, it is hard to distinguish between the first-order effects of variations of skin and skull conductivity and the second-order skin-skull sensitivity in a sensitivity study, but the practical implications are the same.

Whereas prior studies investigated the sensitivity of the EEG forward solution toward conductivity variations only for a few sources, we present results for sources positioned on the whole cortex surface and variations of four tissue conductivity in our study. Vallaghé and Clerc (2008) and Vorwerk et al. (2019a) both analyzed a source in the postcentral gyrus, for which it is not directly clear in how far the results are representative for general source positions. Our study shows that the results for such a source can indeed be generalized for most sources on sulcal walls, whereas we find a slightly different sensitivity distribution for sources on top of gyri and at the bottom of sulci. For such sources, the sensitivity toward skin and skull conductivity may be reduced and a higher sensitivity toward variations of gray and white matter conductivities can be found. This corresponds to the results of Gençer and Acar (2004), who found a strong dependency of the sensitivity values on the dipole direction.

### 4.2 EEG inverse problem

We find the strongest influence of conductivity variations for sources inside of sulci, especially on sulcal walls, and in the longitudinal fissure on EEG source localizations. For these sources, we observe a strong change of the depth of the source reconstruction as a result of conductivity variations (Figure 7). We find average localization errors of up to 1 cm, which corresponds to an extent of the point cloud of source localizations of up to 2 cm. A large amount of these localization errors is caused by incorrect reconstructions of the source depth, i.e., the sources are localized more superficial or deeper on the sulcal wall than the original source position, which makes these mislocalizations relatively predictable. The localization of sources on top of gyri, which mostly have a quasi-radial or partially quasi-radial source orientation, is clearly less affected by conductivity variations. However, only a small amount of these localization errors is caused by an incorrect reconstruction of the source depth, and thus has to be mainly caused by mislocalizations in a direction tangential to the inner skull surface, which could be a mislocalization of the source position along the top of the gyri or to the top of a neighboring gyri. In consequence, this makes these mislocalizations—if they occur—potentially harder to predict than those for quasi-tangential sources.

At all source depths except for very deep brain areas, we find a strong correlation between the localization error and the change in skull conductivity (Figure 10). Investigating the correlation between tissue conductivity variations and the change in source depth, we find a strong negative correlation with the skull conductivity and a positive correlation with the skin conductivity. This means that underestimating the skull conductivity leads to a too shallow source reconstruction and overestimating to a too deep source reconstruction. The opposite effect is found for the skin conductivity. Changes in gray and white matter conductivity neither affected the general localization error nor the source depth.

Our results are in line and expand upon prior studies investigating the influence of tissue conductivity variations on EEG source localizations (Vanrumste et al., 2000; Chen et al., 2010; Akalin Acar and Makeig, 2013; Aydin et al., 2014; Vorwerk et al., 2019a; McCann and Beltrachini, 2022). These studies mostly focused on the effect of variations of the skull conductivity or only investigated single source positions. Our study shows that the effects of skin conductivity variations on the depth of source reconstructions found in Vanrumste et al. (2000) and Aydin et al. (2014) can be generalized for almost all source positions with limitations for very superficial sources. Furthermore, our study confirmed the opposing effects of variations of skin and skull conductivity and confirmed the effect of the skin conductivity on the depth of the reconstructed source found in Vorwerk et al. (2019a) for general source positions. Finally, we also found that the effects of gray and white matter conductivity variations on source localizations remain negligible even for very deep cortical sources, e.g., in the insula.

### 4.3 Limitations

To obtain results that are universally applicable, we made use of a head model based on an averaged MRI template in this study. Therefore, any effects due to individual anatomical variations should be excluded. The stability of our results over the whole cortex suggests that these can largely be transferred to individual head models, of course, except in cases with significant variations of the anatomy such as skull openings, brain resections, or lesions (Oostenveld and Oostendorp, 2002; Brodbeck et al., 2009; Rullmann et al., 2009; Lanfer et al., 2012).

The head model used in this study has two major simplifications compared to six-layer state-of-the-art head models with anisotropic white matter conductivity. We did not include white matter anisotropy in our study, as no such data are available for the New York Head. Given the small influence of variations of the white matter conductivity found for nearly all source positions in this study, it can be assumed that this simplification did not have any significant effect on the outcome of our study. Furthermore, we did not include the distinction between skull compacta and spongiosa, but modeled a homogeneous skull compartment instead. Prior studies have shown that neglecting this distinction can especially affect the accuracy of the EEG forward solution in temporal regions (Vorwerk et al., 2014; Nielsen et al., 2023), below suture lines (McCann and Beltrachini, 2022), or at the skull base (Montes-Restrepo et al., 2014). Since we did not consider this distinction in both the forward and inverse calculations, there should be no direct impact on the results of this study. However, considering variations of skull compacta and spongiosa conductivities separately would add another layer of complexity and might be of interest in future studies. To keep the computational complexity within bounds one might neglect variations of gray and white matter conductivities in turn, which were found to have only a minor influence in our study.

Both in the forward and inverse studies, we focused on single dipole scenarios, i.e., extended source models were not investigated. In general, the results of our inverse study should translate for all inverse methods that allow for an accurate localization of single dipoles. This includes not only dipole scans and dipole fits, but also beamforming methods (Sekihara and Nagarajan, 2008; Westner et al., 2022) and some current-density reconstruction methods, e.g., Bayesian methods (Lucka et al., 2012; Costa et al., 2017; Rezaei et al., 2020), minimum norm estimates (MNE) with depth weighting (Fuchs et al., 1999), or LORETA variations (Pascual-Marqui et al., 2002). Contrary to this, Stenroos and Hauk (2013) have shown that classical MNEs are robust against skull conductivity errors. However, this comes at the cost of an increased localization error for sources that are not superficial (Stenroos and Hauk, 2013, Figure A1), since MNE suffers from depth-bias, i.e., the peak of the reconstructed current density is generally localized too superficial for deep sources (Fuchs et al., 1999; Lucka et al., 2012). Thus, MNEs are not a suitable inverse approach in scenarios where deeper sources are assumed. In other scenarios, e.g., group studies in which widespread brain activation is reconstructed and compared between subjects, the benefit of the robustness against skull conductivity variations might outweigh the disadvantage of the depth-bias. In general, the effect of conductivity variations on the reconstruction of extended sources was not investigated in this study. However, it can be assumed that our results can be generalized to such cases as long as the source is still predominantly dipolar.

Finally, it has to be noted that our choice of the intervals within which the tissue conductivities may vary (Table 1) represents a worst-case scenario. Not only were these intervals chosen rather widely, but it can also be assumed that the real distribution of the conductivities is not uniform but more focused around the literature value (McCann et al., 2019). In practice, the tissue conductivity uncertainties could potentially be more realistically modeled through, e.g., β- or Normal distributions (Gutiérrez et al., 2004; Saturnino et al., 2019). However, these distributions require additional parameters, which, again, are not known a priori and need to be estimated based on the literature.

## 5 Conclusion

In this study, we found that the topography of EEG forward solutions for source positions on the whole cortex surface is mostly sensitive to variations of skull and skin conductivity. The magnitude of EEG forward solutions is also very sensitive to the skull conductivity, but almost similarly sensitive to the gray matter conductivity and to a smaller degree also to the skin conductivity. Analyzing the EEG inverse problem, we find that these changes in the EEG forward solutions translate to localization errors particularly for sources inside of sulci, with the strongest effect on sources on the sulcal walls. For these sources, the localization errors clearly correlate with variations in skull and skin conductivity resulting in changes in the source depth of the reconstructed sources. Sources on top of gyri showed the strongest topography changes for varying tissue conductivities, but these changes resulted in smaller source reconstruction errors than for sources inside of sulci. We are convinced that these results help to better estimate the uncertainty inherent to EEG source localizations. Furthermore, our study shows the additional value of skull conductivity calibration, as the inter-individual variation of the skull conductivity is one of the main sources of EEG source analysis uncertainties affecting almost all cortex areas.

## Data availability statement

The datasets presented in this study can be found in online repositories. The names of the repository/repositories and accession number(s) can be found below: https://www.parralab.org/nyhead/.

## Author contributions

JV: Conceptualization, Funding acquisition, Investigation, Methodology, Writing – original draft, Writing – review & editing. CW: Conceptualization, Funding acquisition, Supervision, Writing - review & editing. DB: Conceptualization, Funding acquisition, Resources, Supervision, Writing - review & editing.
