# Supplementary material for: Global sensitivity of EEG source analysis to tissue conductivity uncertainties
Source: Front Hum Neurosci. 2024 Mar 12;18:1335212. doi: 10.3389/fnhum.2024.1335212 (PMC10963400; doi:10.3389/fnhum.2024.1335212)
Supplement: Supplementary file 1 [file Data_Sheet_1.PDF]

Supplementary Material

1 CLOSED SOBOL INDICES

If we separate the set of parameters $\mathbf{x} = (x_1, \ldots, x_n)$ with indices $\{1, \ldots, n\}$ in two groups $\mathbf{p} = \{i_1, \ldots, i_s\} \subset \{1, \ldots, n\}$ and $\mathbf{q} = \{1, \ldots, n\} \backslash \mathbf{p}$, we denote by $S^{\mathbf{p}}$ the *closed sensitivity index* or closed Sobol index (Janon et al., 2014). This index summarizes all the single effects as well as interactions of the parameters indicated in $\mathbf{p}$, but no interactions with elements of $\mathbf{q}$. In the case $\mathbf{q} = \{i\}$, i.e., $|\mathbf{q}| = 1$, $S^{\mathbf{p}}$ is exactly the first-order Sobol index S_i.

Based on the closed Sobol indices, it is now possible to calculate the higher-order Sobol indices, e.g., in the case of second-order Sobol indices:

$$S_{i,j} = S^{\{i,j\}} - S^{\{i\}} - S^{\{j\}} = S^{\{i,j\}} - S_i - S_j, \tag{S1}$$

i.e., we can calculate the second-order Sobol index $S_{i,j}$ by subtracting the two first-order indices S_i and S_j from the closed Sobol index $S^{\{i,j\}}$. Furthermore, we can calculate the total Sobol index S_i^T via

$$S_i^T = 1 - S^{\mathbf{p}} \tag{S2}$$

with $\mathbf{p} = \{1, \ldots, n\} \backslash \{i\}$. Since the closed Sobol index $S^{\mathbf{p}}$ contains all effects and interactions of parameters not including parameter i, the difference to 1 is exactly all the effects and interactions involving parameter i.

Thus, being able to estimate the closed Sobol indices would allow us to derive both total Sobol indices and arbitrary first- and higher-order indices.

2 JANON ESTIMATOR

We consider a function $Y = f(\mathbf{X}) = f(\mathbf{X}^{\mathbf{p}}, \mathbf{X}^{\mathbf{q}})$ depending on n independent random variables X_i that are separated according to the disjunct sets $\mathbf{p}$ and $\mathbf{q}$ as defined previously. Following Janon et al. (2014), the closed Sobol index $S^{\mathbf{p}}$ can now be written as

$$S^{\mathbf{p}} = \frac{\mathbb{V}_{\mathbf{p}}[\mathbb{E}_{\mathbf{q}}[Y|\mathbf{X}^{\mathbf{p}}]]}{\mathbb{V}[Y]}, \tag{S3}$$

where the expectation value $\mathbb{E}_{\mathbf{q}}$ is calculated with regard to the parameter set $\mathbf{q}$ and the variance $\mathbb{V}_{\mathbf{p}}$ is calculated with regard to the parameter set $\mathbf{p}$. Heuristically this means that the influence of the paramter set $\mathbf{q}$ is integrated out when taking the expectation value, whereas the influence of the parameter set $\mathbf{p}$ is then determined by calculating the variance.

To be able to actually calculate $S^{\mathbf{p}}$, it is useful to express it in terms of covariances (Janon et al., 2014):

$$S^{\mathbf{p}} = \frac{\operatorname{cov}(Y, Y^{\mathbf{p}})}{\mathbb{V}(Y)}, \tag{S4}$$

with $Y^{\mathbf{p}} = f(\mathbf{X}^{\mathbf{p}}, \mathbf{X}'^{\mathbf{q}})$, where $\mathbf{X}'^{\mathbf{q}}$ denotes an independent copy of $\mathbf{X}^{\mathbf{q}}$.

The formulation in terms of covariances allows to derive different estimators for $S^{\mathbf{p}}$, such as the *Homma estimator* (Homma and Saltelli, 1996) and the *Janon estimator* (Janon et al., 2014) that has been used in this manuscript:

$$S_N^{\mathbf{p}} = \frac{\frac{1}{N}\sum y_i y_i^{\mathbf{p}} - \left(\frac{1}{N}\sum\left[\frac{y_i + y_i^{\mathbf{p}}}{2}\right]\right)^2}{\frac{1}{N}\sum\left[\frac{y_i^2 + (y_i^{\mathbf{p}})^2}{2}\right] - \left(\frac{1}{N}\sum\left[\frac{y_i + y_i^{\mathbf{p}}}{2}\right]\right)^2}, \tag{S5}$$

where y_i and $y_i^{\mathbf{p}}$, $i = 1 \ldots, N$ are realizations of $Y = f(\mathbf{X}^{\mathbf{p}}, \mathbf{X}^{\mathbf{q}})$ and $Y^{\mathbf{p}} = f(\mathbf{X}^{\mathbf{p}}, \mathbf{X}'^{\mathbf{q}})$, respectively. The Janon estimator was shown to have optimal asymptotic variance and is robust against model perturbations (Janon et al., 2014).

For further details, we would like to refer the reader to the the original publication by Janon et al. (2014) and the UQLab User Manual (`https://uqftp.ethz.ch/uqlab_doc_pdf/2.0.0/UserManual_Sensitivity.pdf`), particularly Sec. 1.5.3.2 “Sensitivity Indices” and 2.1.8 “MC-based Sobol Indices”.

REFERENCES

Homma, T. and Saltelli, A. (1996). Importance measures in global sensitivity analysis of nonlinear models. *Reliability Engineering & System Safety* 52, 1–17

Janon, A., Klein, T., Lagnoux, A., Nodet, M., and Prieur, C. (2014). Asymptotic normality and efficiency of two sobol index estimators. *ESAIM: Probability and Statistics* 18, 342–364
